# Correlates of chronic pain onset and recovery in the CoLaus cohort

**DOI:** 10.1002/ejp.4712

**Published:** 2024-08-07

**Authors:** Giada Dirupo, Jean‐Benoît Rossel, Nicolas Fournier, Audrey D'Andrea, Peter Vollenweider, Isabelle Decosterd, Marc René Suter, Chantal Berna

**Affiliations:** ^1^ Center for Integrative and Complementary Medicine, Department of Anesthesiology Lausanne University Hospital (CHUV), The Sense and University of Lausanne Lausanne Switzerland; ^2^ Department of Clinical Neurosciences, Laboratory for Research in Neuroimaging (LREN) Lausanne University Hospital (CHUV) and University of Lausanne Lausanne Switzerland; ^3^ Center for Primary Care and Public Health (Unisanté) University of Lausanne Lausanne Switzerland; ^4^ Pain Center, Department of Anesthesiology Lausanne University Hospital (CHUV) and University of Lausanne Lausanne Switzerland; ^5^ Department of Medicine, Internal Medicine Lausanne University Hospital (CHUV) and University of Lausanne Lausanne Switzerland; ^6^ Department of Fundamental Neurosciences, Faculty of Biology and Medicine (FBM) University of Lausanne Lausanne Switzerland

## Abstract

**Background:**

Only few previous cohort studies examined simultaneously predictors of chronic pain (CP) onset and recovery. Furthermore, these studies used various sociodemographic and pain‐related characteristics, without standardized measures of sleep and depression. The present study aimed at expanding and strengthening these findings in a large Swiss population.

**Methods:**

We analysed data from a longitudinal cohort (*n* = 4602) collected at two time points separated by 5 years in Lausanne, Switzerland. We studied through two independent multivariable logistic regression models, the predictors of CP onset and recovery, including socio‐demographic data as well as standardized measures of sleep and mood.

**Results:**

Chronic pain was reported by 43.1% and 44.4% of participants, with 11.6% at the second follow‐up reporting moderate or intense pain. Neuropathic pain, regardless of intensity, had a more negative impact on quality of life. An inferential model (*n* = 1331) identified the male sex as predictive for recovering from CP. Older age, being overweight or obese (compared to normal weight), higher depression scores and pain medication intake were predictive for sustained pain at the second follow‐up. A second model (*n* = 1886) identified being overweight or obese (compared to normal weight), low quality of sleep and being a former smoker (compared to a non‐smoker) as predictive for developing CP, while the male sex was lowering the risk.

**Conclusions:**

While sex and weight are associated with both recovery and new CP onset, separate variables also need to be considered in these processes, underlining specific factors to be addressed, depending on the context, whether preventive or therapeutic.

**Significance Statement:**

Multivariable models in a Swiss cohort (*N* = 4602) associate male sex, not taking pain medication, normal weight, lower depression scores and younger age with recovery from chronic pain, while females, obese or overweight, having worse sleep and former smokers are associated with onset of new chronic pain. These common and separate factors need to be considered in treatment and prevention efforts.

## 1 INTRODUCTION

Predictors of chronic pain (CP) recovery and development are crucial for improving healthcare policies and prevention efforts (Hagen et al., 2020; Larsson et al., 2017; Samuelsen et al., 2016; van Hecke et al., 2013; VanDenKerkhof et al., 2016) and have inspired new studies of pain trajectories (Picavet et al., 2019). One key question is whether the risk factors for developing pain are the same in reverse for its recovery. Surprisingly, only few large cohort‐based studies have focused on predictors of recovery from CP. One British study (*n* = 2184) identified younger age, lower bodily pain and better general health (Elliott et al., 2002). A Danish study (*n* = 2649), identified younger age, male sex, living with someone, good self‐rated general health, good mental health, having no diseases and a higher income (Eriksen et al., 2004). Another Danish study (*n* = 2354) underlined the absence of opioid intake (Sjøgren et al., 2010). Furthermore, in a Dutch study (*n* = 3412), CP recovery was more likely in people with better perceived general and mental health and without chronic musculoskeletal complaints (Picavet et al., 2019). These studies also examined risk factors for developing CP, confirming separate findings from large databases (e.g. the UK Biobank), which can be grouped as psychological (depression, loneliness, social exclusion, lower social functioning), sociodemographic (being female, being retired) and health‐related (type II diabetes, previous fractures, lower physical functioning) (Carvalho et al., 2020; Macfarlane et al., 2015; Mills et al., 2019). Nevertheless, crucially, in the studies examining both recovery and new CP, important variables such as quality of sleep and depression were not consistently assessed through validated questionnaires. Instead, for example, the most recent trajectory study (Picavet et al., 2019) used a subjective measure of mental health with a dichotomous cut‐off as well as a self‐rated number of hours of sleep (an index that is subject to biases and poorly correlated with sleep quality (Girschik et al., 2012)). Hence, there is an interest in increasing the body of evidence regarding pain trajectories over the course of a 5‐year period in a new population including validated measures of mood and sleep quality.

The well‐qualified pain characteristics allowed a secondary question concerning the impact of neuropathic pain, (i.e. pain due to a lesion of the somatosensory system). Neuropathic pain concerns about one‐third of people with chronic pain (van Hecke et al., 2014). It is often refractory to treatment or under‐treated (Torrance et al., 2013). Neuropathic pain seems to have a larger impact on the patients' lives than other types of chronic pain, regardless of intensity (Attal et al., 2011; Toth et al., 2009), yet given the societal impact, these results deserve replication in our large cohort.

In sum, this study aimed to investigate in a large Swiss Cohort the predictors for recovery and development of CP considering validated clinical measures of depression and sleep quality as well as standard socio‐demographical and biological variables. A secondary question was to evaluate the impact of neuropathic pain on quality of life, adjusted for pain intensity.

## 2 METHODS

### 2.1 Participants

**FIGURE 1:**
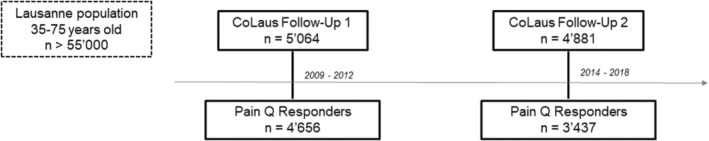
CoLaus cohort inclusion chart. Participants were included from a sample of the Lausanne (Switzerland) population of people aged 35–75 years. The number of participants at each data collection timepoint is shown in the rectangles above the timeline. Pain questionnaires (Pain Q) were collected on a voluntary basis at Follow‐ups 1 and 2 (FU1, FU2). The rectangles below the timeline present the number of responders to the Pain Q.

This sample is part of the CoLaus longitudinal cohort, which included residents aged 35–75 years of the city of Lausanne, Switzerland (see Firmann et al., 2008 for details on the recruitment procedure). Briefly, all participants signed informed consent before the first data collection (baseline, *n* = 6734, not used here) that took place between 2003 and 2006 and were then invited for a first (FU1, *n* = 5064) and second follow‐up (FU2, *n* = 4881), respectively, 5 and 10 years later. As part of both follow‐ups (but not the baseline assessment), sub‐questionnaires regarding pain were collected. The response to all CoLaus sub‐questionnaires was optional, and a proportion of the participants did not fill the pain questionnaires (details in Figure 1). The institutional Ethics Committee of the University of Lausanne, which then became the Ethics Commission of Canton Vaud (www.cer‐vd.ch) approved the baseline CoLaus‐PsyCoLaus study (project number PB_2018‐00038, reference 239/09) and the follow‐ups (respectively, reference 33/09, decision of 23 February 2009 and reference 26/14, decision of 11 March 2014). The study was performed in agreement with the Helsinki declaration, and in accordance with the applicable Swiss legislation (LRH 810.30, approved by the Swiss Federal Parliament on 30 September 2011). Sub‐samples of the CoLaus cohort that also completed specific psychiatric investigations have allowed studies of pain with a geriatric focus (Rouch et al., 2021) and of psychiatric predictors of pain (Rouch et al., 2023).

#### 2.1.1 Socio‐demographic and health assessment

All CoLaus participants filled questionnaires sent per post to their homes and came to a medical visit, where questions were clarified. They provided detailed demographic information, such as sex, age, family situation and socio‐economic status, smoking status (never (=non‐smoker), current, former) and self‐rated general health (‘How would you rate your general health?’ coded from 1 (best) to 5 (worst)) (Firmann et al., 2008); height and weight were measured during the medical visit and allowed to calculate BMI, which was classified as normal, overweight or obese; fasting blood samples were collected and allowed to determine the glycaemia, with clinical cut‐offs for diabetes diagnosis. Depression was assessed with the Center for Epidemiologic Studies Depression Scale (CES‐D) score (Morin et al., 2011). Cognitive functions were assessed through the Mini‐Mental State Examination (MMSE) (Derouesne et al., 1999). Finally, sleep quality was assessed with the Pittsburgh Sleep Quality Index (PSQI) (Ait‐Aoudia et al., 2013), which produces a value ranging from 0 (no difficulties) to 21 (worst sleep quality).

#### 2.1.2 Pain and related measures

The presence of CP at a given follow‐up was defined by a positive answer to two screening questions: ‘Do you currently suffer from pain on a daily basis in one or multiple locations?’ (Y/N) and ‘Since when do you suffer from daily pain?’ (>3 months Y/N) (Bouhassira et al., 2008). This combination of factors led to a variable ‘chronic pain’ (present/absent; CP +/−) at each follow‐up.

If daily pain was reported, further questions were asked regarding pain locations (yes/no for a list of all body parts) and the most painful area (choice of only one body part, Figure S1)

At Follow‐up 2, additional questions assessed (a) the intensity of pain (maximum, minimum and average over the past week on a numerical rating scale ranging from 0 = minimum intensity to 10 = maximum intensity) (Bouhassira et al., 2008) and (b) neuropathic characteristics with the self‐reported DN4 score (Bouhassira et al., 2005).

#### 2.1.3 Pain‐relevant medications

Participants indicated medications intake, which were then grouped into the following pain‐relevant categories: opioids, non‐steroidal anti‐inflammatory drugs (NSAID) and paracetamol, gabapentinoids, antidepressants with pain‐modulating effects (tricyclics & noradrenaline and serotonin reuptake inhibitors [NSRI]). Two categorical variables were created: ‘pain medications’ (opioids + NSAID and paracetamol) and ‘pain medication adjuvants’ (gabapentinoids + antidepressants with pain modulating effects) with a 1/0 code based on taking or not at least one medication in either of these categories (see Table S1 for the list of medications).

### 2.2 Statistical approach

#### 2.2.1 Descriptive analyses

Socio‐demographic and health characteristics were chosen based on previous studies (Mills et al., 2019), namely, sex, age, work status, marital status, self‐rated general health, quality of sleep and depression. We first compared these characteristics for responders vs. non‐responders to the pain questionnaire (see Table 1A and Table 1B) to assess how representative our sample was of the full cohort through Student's t‐tests and chi‐squared tests for continuous and categorical variables, respectively. We computed OR and Cohen's *d* to further investigate significant group differences and report in text the large effects. We described the population included at both follow‐ups (Table 2) reporting the number and percentage of participants with their characteristics, according to the absence or the presence of CP.

**TABLE 1A:** Characteristics of the responders and non‐responders at FU1.

Variable	Non‐responders (*N =* 462)	Responders (*N =* 4602)	*p*‐value
Sex			
Woman	223 (48.3%)	2484 (54.0%)	0.019
Man	239 (51.7%)	2118 (46.0%)	
Age (years)—mean (SD)	58 (10.9)	57.7 (10.5)	0.566
Working			
Yes	212 (51.7%)	2603 (56.8%)	0.046
No	198 (48.3%)	1979 (43.2%)	
Missing	52	20	
Living situation			
Living alone	144 (35.4%)	1270 (27.6%)	<0.001
Single‐parent family	32 (7.9%)	253 (5.5%)	
Couple without children	115 (28.3%)	1642 (35.7%)	
Couple with children	116 (28.5%)	1431 (31.1%)	
Missing	55	6	
How would you rate your health (1–5)			
Mean (SD)	2.2 (0.8)	1.9 (0.7)	<0.001
Missing	7	20	
How would you rate your health (cat)			
Very good	70 (15.4%)	1149 (25.1%)	<0.001
Good	249 (54.7%)	2696 (58.8%)	
Average	101 (22.2%)	677 (14.8%)	
Bad	28 (6.2%)	54 (1.2%)	
Very bad	7 (1.5%)	6 (0.1%)	
Missing	7	20	
Sleep: PSQI (0–21)			
Mean (SD)	5.7 (3.5)	5.0 (3.2)	0.109
Missing	408	578	
Depression: CES‐D score (0–60)			
Mean (SD)	11.6 (9.5)	10.7 (8.7)	0.461
Missing	408	354	
Mini‐Mental Examination (MME) score			
Mean (SD)	27.9 (2.8)	29.2 (1.6)	<0.001
Missing	302	2767	

Abbreviations: CES‐D, Center for Epidemiologic Studies Depression Scale; PSQI, Pittsburgh Sleep Quality Index.

**TABLE 1B:** Characteristics of the responders and non‐responders at FU2.

Variable	Non‐responders (*N* = 1444)	Responders (*N* = 3437)	*p*‐value
Sex			
Woman	825 (57.1%)	1864 (54.2%)	0.063
Man	619 (42.9%)	1573 (45.8%)	
Age (years)—mean (SD)	65.6 (11.0)	61.8 (10.0)	<0.001
Working			
Yes	463 (39.1%)	1967 (57.8%)	<0.001
No	721 (60.9%)	1436 (42.2%)	
Missing	260	34	
Living situation			
Living alone	468 (40.2%)	971 (28.7%)	<0.001
Single‐parent family	49 (4.2%)	199 (5.9%)	
Couple without children	403 (34.6%)	1275 (37.7%)	
Couple with children	245 (21.0%)	939 (27.7%)	
Missing	*279*	53	
How would you rate your health (1–5)			
Mean (SD)	2.2 (0.8)	2.0 (0.7)	<0.001
Missing	*41*	9	
How would you rate your health (cat)			
Very good	215 (15.3%)	817 (23.8%)	<0.001
Good	766 (54.6%)	1945 (56.7%)	
Average	363 (25.9%)	603 (17.6%)	
Bad	50 (3.6%)	60 (1.8%)	
Very bad	9 (0.6%)	3 (0.1%)	
Missing	*41*	9	
Sleep—PSQI (0–21)			
Mean (SD)	4.9 (2.9)	4.9 (3.1)	0.932
Missing	*1287*	1008	
Depression: CES‐D score (0–60)			
Mean (SD)	11.6 (8.5)	10.0 (8.4)	0.002
Missing	1161	178	
Mini‐Mental Examination (MME) score			
Mean (SD)	28.3 (2.7)	29.1 (2.1)	<0.001
Missing	813	1769	

*Note*: Participants were invited (yet not obliged) to fill a series of questionnaires, including those on pain. Here we show the characteristics of the subgroups of participants who responded to demographic questionnaires but not the pain one (non‐responders) and those who responded to both (responders) at follow‐ups (FU) 1 (Table 1A) and 2 (Table 1B). The last columns indicate the p‐values of the chi‐squared tests or Student's *t*‐tests expressing the difference between groups. For all these statistically significant differences, the effect sizes are low or moderate (ORs ≤ 1.28; Cohen's *d* ≤ 0.37) except for the higher proportion of working individuals in responders (OR = 2.13, 95% CI = 1.86–2.44).

**TABLE 2:** Characteristics of the samples used in the descriptive analyses.

Variable	FU1			FU2		
	CP− (*N* = 2617)	CP+ (*N* = 1985)	*p*‐value	CP− (*N* = 1912)	CP+ (*N* = 1525)	*p*‐value
Sex						
Woman	1306 (49.9%)	1178 (59.3%)	<0.001	936 (49.0%)	928 (60.9%)	<0.001
Man	1311 (50.1%)	807 (40.7%)		976 (51.0%)	597 (39.1%)	
Age (years)—Mean (SD)	56.4 (10.3)	59.5 (10.5)	<0.001	60.9 (9.8)	63.0 (10.2)	<0.001
Age category						
≤50	871 (33.3%)	454 (22.9%)	<0.001	293 (15.3%)	174 (11.4%)	<0.001
>50 and ≤60	812 (31.0%)	576 (29.0%)		703 (36.8%)	484 (31.7%)	
>60 and ≤70	630 (24.1%)	600 (30.2%)		509 (26.6%)	430 (28.2%)	
>70	304 (11.6%)	355 (17.9%)		407 (21.3%)	437 (28.7%)	
Living situation						
Living alone	699 (26.7%)	571 (28.8%)	<0.001	511 (27.1%)	460 (30.7%)	0.009
Single‐parent family	139 (5.3%)	114 (5.8%)		99 (5.2%)	100 (6.7%)	
Couple without children	882 (33.7%)	760 (38.3%)		721 (38.2%)	554 (37.0%)	
Couple with children	894 (34.2%)	537 (27.1%)		556 (29.5%)	383 (25.6%)	
Missing	3	3		25	28	
Working						
Yes	1650 (63.4%)	953 (48.2%)	<0.001	1175 (62.0%)	792 (52.5%)	<0.001
No	954 (36.6%)	1025 (51.8%)		719 (38.0%)	717 (47.5%)	
Missing	13	7		18	16	
Sleep—PSQI (0–21)						
Mean (SD)	4.4 (2.8)	5.8 (3.5)	<0.001	4.3 (2.7)	5.7 (3.4)	<0.001
Missing	302	276		507	501	
Depression: CES‐D score (0–60)						
Mean (SD)	9.3 (7.9)	12.7 (9.4)	<0.001	8.4 (7.4)	12.0 (9.1)	<0.001
Missing	176	178		92	86	
MMSE						
Mean (SD)	29.2 (1.5)	29.1 (1.7)	0.082	29.1 (2.1)	29.1 (2.1)	0.891
Missing	1709	1058		1052	717	

The number of participants is indicated for each FU and subgroups reporting CP (CP+) or not (CP‐). The last column indicates the p‐values of the chi‐squared tests or Student's *t*‐tests expressing the difference between groups.

Abbreviation: MMSE, Mini‐mental state examination score.

Finally, for FU2, we compared the characteristics of CP+ sub‐groups according to pain intensity (Table S2) and neuropathic vs. non‐neuropathic characteristics (Table S3). Furthermore, we investigated the impact of the type of pain (neuropathic vs non‐neuropathic), adjusted for pain intensity, on depression, quality of sleep and self‐rated general health by computing three independent linear models (one for each variable).

#### 2.2.2 Predictor analyses

We computed two independent analyses, aimed at identifying potential predictors of recovering from CP and developing it, respectively. The statistical approach we chose was previously used in large dataset studies (e.g. Liu et al., 2020) and consisted of two steps: 1) variables selection (through univariate models) and 2) a multivariable model. The two steps were realized through independent analyses for the two clinical paths of interest (i.e. recovering from CP and developing CP) following the exact same procedure. These two analysis steps are described in more detail below.

##### Variables selection—Univariate analysis

First, we created one dichotomic variable for subjects who recovered from CP (who had pain at FU1 and not at FU2), which we named ‘recovered from chronic pain’ (RCP). People with pain at both FUs were the comparator. We then regressed it against each variable (at FU1) individually by an independent logistic regression model with a significance level set at 0.05. This led to a list of variables significantly associated with recovering from CP.

We also created a variable for subjects that had no pain at FU1 and developed this condition at FU2 called ‘developed chronic pain’ (DCP) and repeated the same logistic regressions with this dependent variable. Here, people without pain at both FUs acted as comparators.

To avoid biases or non‐reliable results, only the variables with at least 80% valid data were considered. We therefore discarded the MMSE and the pain locations. See Table 3A and Table 3B for a list of the included factors and the significant results of the univariate models, and Tables S7 and S8 for details on the missing data.

**TABLE 3A:** Univariate analyses of the chances of recovering from chronic pain.

Variable	Odds ratio (95% CI; *p*‐value)
Sex	
Woman	1 (ref.)
Man	**1.435** (**1.105–1.864**; ***p* = 0.007**)
Age (in decades)	**0.809** (**0.710–0.921**; ***p* = 0.001**)
How would you rate your health	**0.586** (**0.478–0.719**; ***p* < 0.001**)
Felt tired	**0.685** (**0.560–0.838**; ***p* < 0.001**)
Difficulties in taking a shower or dressing up	**0.602** (**0.406–0.894**; ***p* = 0.012**)
Difficulties in going shopping or doing household tasks	**0.414** (**0.268–0.639**; ***p* < 0.001**)
CES‐D overall score	**0.962** (**0.946–0.977**; **p < 0.001**)
PSQI	**0.926** (**0.890–0.964**; **p < 0.001**)
BMI category	
Normal	1 (ref.)
Overweight	**0.672** (**0.506–0.892**; ***p* = 0.006**)
Obese	**0.533** (**0.364–0.782**; **p = 0.001**)
Smoking status	
Non‐smoker	1 (ref.)
Former smoker	0.823 (0.616–1.100; *p* = 0.187)
Current smoker	0.903 (0.637–1.281; *p* = 0.568)
Alcohol (yes vs no)	**1.438** (**1.003–2.060**; ***p* = 0.048**)
Social financial help (yes vs no)	**0.591** (**0.451–0.775**; **p < 0.001**)
Working (yes vs no)	**1.370** (**1.055–1.780**; ***p* = 0.018**)
Living situation	
Living alone	1 (ref.)
Single‐parent family	1.012 (0.570–1.799; *p* = 0.967)
Couple without children	1.263 (0.897–1.779; *p* = 0.182)
Couple with children	**1.588** (**1.114–2.263**; ***p* = 0.010**)
Pain medications (yes vs no)	**0.300** (**0.179–0.504**; **p < 0.001**)
Pain medications adjuvants (yes vs no)	0.706 (0.326–1.530; *p* = 0.378)
Diabetes (yes vs no)	0.711 (0.448–1.128; *p* = 0.147)
Antidiabetics treatment (yes vs no)	0.773 (0.419–1.425; *p* = 0.409)

*Note*: In bold are the significant results, included in the multivariable model (see Figure 2, upper panel).

Abbreviations: CES‐D, center for epidemiologic studies depression scale; PSQI, Pittsburgh sleep quality index.

**TABLE 3B:** Univariate analyses of the risk of developing chronic pain.

Variable	Odds ratio (95% CI; p‐ value)
Sex	
Woman	1 (ref.)
Man	**0.672** (**0.536–0.844**; ***p* = 0.001**)
Age, in decades	1.064 (0.945–1.197; *p* = 0.306)
How would you rate your health	**1.450** (**1.200–1.751**; **p < 0.001**)
Felt tired	**1.423** (**1.188–1.704**; **p < 0.001**)
Difficulties in taking a shower or dressing up	1.440 (0.777–2.670; *p* = 0.247)
Difficult in going shopping or doing household tasks	**1.691** (**1.058–2.701**; ***p* = 0.028**)
CES‐D overall score	**1.025** (**1.011–1.039**; **p = 0.001**)
PSQI	**1.092** (**1.049–1.136**; **p < 0.001**)
BMI category	
Normal	1 (ref.)
Overweight	**1.360** (**1.068–1.731**; ***p* = 0.012**)
Obese	1.426 (0.986–2.063; *p* = 0.060)
Smoking status	
Non‐smoker	1 (ref.)
Former smoker	**1.313** (**1.020–1.689**; ***p* = 0.034**)
Current smoker	1.269 (0.932–1.728; *p* = 0.130)
Do you currently drink alcohol (yes vs no)	0.983 (0.703–1.376; *p* = 0.922)
Do you receive social help (yes vs no)	1.165 (0.907–1.497; *p* = 0.232)
Currently working (yes vs no)	0.860 (0.674–1.097; *p* = 0.224)
Current living situation	
Living alone	1 (ref.)
Single parent family	0.975 (0.571–1.663; *p* = 0.925)
Couple without children	0.743 (0.548–1.007; *p* = 0.055)
Couple with children	0.895 (0.673–1.191; *p* = 0.446)
Pain medications (yes vs no)	**2.485** (**1.088–5.677**; ***p* = 0.031**)
Pain medications adjuvants (yes vs no)	1.655 (0.681–4.021; *p* = 0.266)
Diabetes (yes vs no)	1.261 (0.821–1.937; *p* = 0.290)
Antidiabetics treatment (yes vs no)	1.212 (0.638–2.302; *p* = 0.557)

*Note*: In bold are the significant results, included in the multivariable model (see Figure 2, lower panel).

Abbreviations: CES‐D, center for epidemiologic studies depression scale; PSQI, Pittsburgh sleep quality index.

**TABLE 4A:** Characteristics of the individuals included in the model recovering from chronic pain (CP).

Variable	Persistent CP (*N* = 688)	Recovered CP (*N* = 347)	*p*‐value
Sex			
Woman	431 (62.6%)	187 (53.9%)	0.007
Man	257 (37.4%)	160 (46.1%)	
Age (years)—mean (SD)	58.6 (10.1)	56.5 (9.9)	0.001
How would you rate your health—mean (SD)	2.1 (0.7)	1.9 (0.6)	<0.001
CES‐D overall score—mean (SD)	13.1 (9.2)	10.2 (8.1)	<0.001
BMI categories			
Normal	268 (39.0%)	175 (50.4%)	0.001
Overweight	285 (41.4%)	125 (36.0%)	
Obese	135 (19.6%)	47 (13.5%)	
Do you currently drink alcohol?			
No	129 (18.8%)	48 (13.8%)	0.047
Yes	559 (81.3%)	299 (86.2%)	
Pain medications			
No	582 (84.6%)	329 (94.8%)	<0.001
Yes	106 (15.4%)	18 (5.2%)	
Pain medications adjuvants			
No	663 (96.4%)	338 (97.4%)	0.376
Yes	25 (3.6%)	9 (2.6%)	
Smoking status			
Former smoker	270 (39.2%)	123 (35.4%)	0.418
Non‐smoker	280 (40.7%)	155 (44.7%)	
Current smoker	138 (20.1%)	69 (19.9%)	
Difficulties in going shopping or doing household tasks—mean (SD)	1.2 (0.4)	1.1 (0.3)	<0.001
Felt tired—mean (SD)	1.7 (0.7)	1.5 (0.6)	<0.001
Difficulties in taking a shower or dressing up—mean (SD)	1.2 (0.4)	1.1 (0.3)	0.011
Social financial help			
No	381 (55.4%)	235 (67.7%)	<0.001
Yes	307 (44.6%)	112 (32.3%)	
Working			
No	333 (48.4%)	141 (40.6%)	0.018
Yes	355 (51.6%)	206 (59.4%)	
Living situation			
Living alone	183 (26.6%)	73 (21.0%)	0.058
Single parent family	52 (7.6%)	21 (6.1%)	
Couple without children	262 (38.1%)	132 (38.0%)	
Couple with children	191 (27.8%)	121 (34.9%)	
Sleep—PSQI—mean (SD)	6.0 (3.6)	5.1 (3.1)	<0.001
Diabetes (WHO criteria)			
No	615 (89.4%)	320 (92.2%)	0.146
Yes	73 (10.6%)	27 (7.8%)	
Antidiabetic drug treatment			
No	650 (94.5%)	332 (95.7%)	0.408
Yes	38 (5.5%)	15 (4.3%)	

*Note*: After exclusion of subjects with incomplete data, the sample is smaller than the responder group.

Abbreviation: BMI, body mass index.

##### Main model—Multivariable analysis

We computed an independent multivariable logistic regression model to identify which variables were associated with a higher likelihood of recovering from CP. This was repeated for identifying the variables associated with developing CP. The models had either RCP or DCP as the dependent variable and the significant variables selected in the previous step (univariate analysis) as predictors. To ensure that we did not have multi‐collinearity issues in our data, we calculated the variance inflation factor (VIF) for each variable in our multivariable models and verified that for none of them, the VIF was above 2.

All analyses were performed with the software Stata, version 17.0 (StataCorp. 2021. Stata Statistical Software: Release 17. College Station, TX: StataCorp LLC.).

## 3 RESULTS

### 3.1 Sample description

#### 3.1.1 Responders vs non‐responders

At FU1 4656 subjects out of 5064 were initially considered responders, but the presence/absence of CP could not be computed for 54 of them due to missing data, resulting in a final sample of 4602 participants (91.9% of the CoLaus Cohort). Similarly, at FU2 (on average 5 years later) 3590 out of 4881 participants were classified as responders but 153 were excluded due to missing data, leading to a sample of 3437 participants (70.4% of the CoLaus Cohort) (see Figure 1 and Table S7).

**TABLE 4B:** Characteristics of the individuals included in the model developing chronic pain (CP).

Variable	Stayed healthy (*N* = 1′110)	Developed CP (*N* = 416)	*p*‐value
Sex			
Woman	512 (46.1%)	233 (56.0%)	<0.001
Man	598 (53.9%)	183 (44.0%)	
Age (years)—mean (SD)	54.6 (9.5)	55.2 (9.4)	0.306
How would you rate your health—mean (SD)	1.7 (0.6)	1.8 (0.6)	<0.001
CES‐D overall score—mean (SD)	8.6 (7.7)	10.1 (8.2)	<0.001
BMI categories			
Normal	595 (53.6%)	190 (45.7%)	0.021
Overweight	403 (36.3%)	175 (42.1%)	
Obese	112 (10.1%)	51 (12.3%)	
Do you currently drink alcohol?			
No	142 (12.8%)	54 (13.0%)	0.922
Yes	968 (87.2%)	362 (87.0%)	
Pain medications			
No	1098 (98.9%)	405 (97.4%)	0.026
Yes	12 (1.1%)	11 (2.6%)	
Pain medications adjuvants			
No	1097 (98.8%)	408 (98.1%)	0.262
Yes	13 (1.2%)	8 (1.9%)	
Smoking status			
Former smoker	394 (35.5%)	167 (40.1%)	0.081
Non‐smoker	511 (46.0%)	165 (39.7%)	
Current smoker	205 (18.5%)	84 (20.2%)	
Difficulties in going shopping or doing household tasks—mean (SD)	1.0 (0.2)	1.1 (0.3)	0.025
Felt tired—mean (SD)	1.4 (0.6)	1.5 (0.6)	<0.001
Difficulties in taking a shower or dressing up—mean (SD)	1.0 (0.2)	1.0 (0.2)	0.245
Social financial help			
No	821 (74.0%)	295 (70.9%)	0.231
Yes	289 (26.0%)	121 (29.1%)	
Working			
No	322 (29.0%)	134 (32.2%)	0.224
Yes	788 (71.0%)	282 (67.8%)	
Living situation			
Living alone	261 (23.5%)	112 (26.9%)	0.258
Single‐parent family	55 (5.0%)	23 (5.5%)	
Couple without children	367 (33.1%)	117 (28.1%)	
Couple with children	427 (38.5%)	164 (39.4%)	
Sleep—PSQI—mean (SD)	4.1 (2.7)	4.8 (2.7)	<0.001
Diabetes (WHO criteria)			
No	1039 (93.6%)	383 (92.1%)	0.289
Yes	71 (6.4%)	33 (7.9%)	
Antidiabetic drug treatment			
No	1079 (97.2%)	402 (96.6%)	0.556
Yes	31 (2.8%)	14 (3.4%)	

*Note*: After exclusion of subjects with incomplete data, the sample is smaller than the responder group.

Abbreviations: BMI, body mass index; CES‐D, center for epidemiologic studies depression scale; PSQI, Pittsburgh sleep quality index; WHO, World Health Organization.

At FU1, the sample of responders was not fully representative of the cohort, showing differences in sex, living situation and self‐rated general health compared to the non‐responders (see Table 1A). Regarding FU2, the subsamples differed in age, working and living status, self‐reported general health and depression scores (see Table 1B). Yet, for all these statistically significant differences, the effect sizes were low or moderate (ORs ≤1.58, Cohen's *d* ≤ 0.45) except for the higher proportion of working individuals in responders at FU2 (OR = 2.13, 95% CI = 1.86–2.44).

#### 3.1.2 Description of the chronic pain population

At FU1 and FU2, respectively, 43.1% and 44.4% of participants reported CP. At FU1, 84.1% of the CP+ sample did not take any medication for pain, 13.6% took only one type of medication and the rest took more than one (two: 2.1%, >2: 0.3%). The most frequently taken medications were NSAIDs and paracetamol (11.5%), antidepressants (tricyclic and NSRI) (3.3%), opioids (2.6%) and finally gabapentinoids (1.0%). At FU2 74.7% of the CP+ sample took no medication, with 20.9% taking only one and the rest taking more than one (two: 3.3%, >2: 1.1%), with NSAID and paracetamol again the most frequent (21.8%), followed by opioids (4.7%), antidepressants (tricyclic and NSRI) (2.5%) and finally gabapentinoids (1.8%) (see Table S4).

At FU1, among CP+ participants, the most painful location was the low back for 19.7%, followed by knees and shoulders (9.5% and 7.3% respectively). Participants most often reported one painful location (29.4%), less often two or more locations in the body (two: 24.4%, three: 15.8%). A similar pattern was observed at FU2 with 16.1% reporting low back as the main source of pain, followed by knees (8.9%) and shoulders (5.5%) (see Figure S1). Out of the full population of responders at FU2, 32.1% reported mild and 11.6% moderate or severe CP. When focusing only on the CP+ participants at FU2 who reported their pain intensity, they were mainly reporting pain of mild intensity (72.2%), with only 19.7% reporting moderate and 6.5% severe pain (see Figure S2). When comparing sample characteristics between mild and moderate/severe intensity, we observed a greater proportion of non‐working people in the latter group (OR = 1.86, CI = 1.46, 2.36). The effect sizes of other significant differences were small when looking at the OR and were not considered relevant (see Table S2 for more details).

#### 3.1.3 Impact of neuropathic pain on quality‐of‐life indicators

At FU2, 24.4% out of CP+ participants who answered the self‐reported DN4 questionnaire qualified for neuropathic pain (see Table S3 for more details). The more intense the pain, the higher the proportion of participants with neuropathic characteristics, with the following percentages: mild: 18.7%, moderate: 37.9% and severe: 48.3% (see Figure S2).

Lower sleep quality (*β* = 0.80, 95% CI = 0.24–1.36, *p* = 0.005), lower self‐rated general health (*β* = 0.29, 95% CI = 0.19–0.39, *p* < 0.001) and more depression (*β* = 3.69, 95% CI = 2.33–5.05, *p* < 0.001) were reported by patients with neuropathic characteristics when controlling for the reported pain intensity.

### 3.2 Inferential analyses

#### 3.2.1 Recovering from CP

**FIGURE 2:**
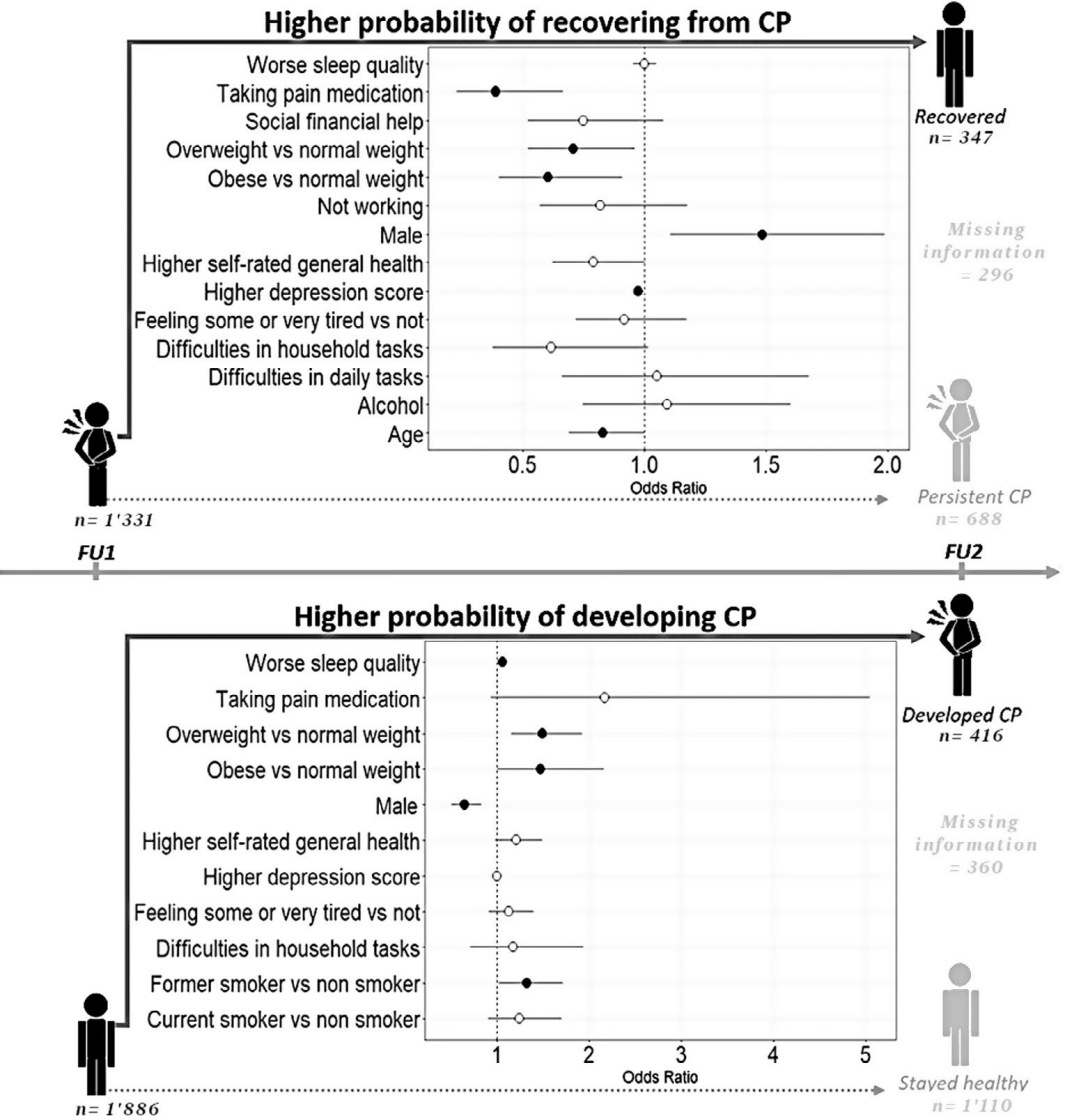
Results of the multivariable models for recovering from or developing chronic pain (CP). Based on the significant variables identified in the univariate models (see Table 4A and 4B), separate multivariable models were computed to identify the predictors of recovering from and developing CP. The odds ratios of variables linked to the chance of recovering from chronic pain at follow‐up 2 (FU2) (upper panel, details in Table S5) and risk of developing chronic pain at FU2 (lower panel, details in Table S6) are represented through empty circles (non‐significant) and black circles (significant). The more at the right a parameter/circle is, the more likely that the variable leads to recovering from/developing CP. The error bars represent the 95% confidence intervals.

Given the exclusion of subjects with missing values the model ultimately ran on a sample of 1331 participants (Figure 2). The variables included in the final model as predictors of recovery from CP (significant results from the univariate analyses) were age, alcohol consumption, BMI, difficulties with household tasks, as well as with daily tasks, depression score, sex, pain medication intake, sleep quality, receiving financial help from the government, whether they were working or not (see Table 3A). The multivariable analysis (details in Table S5) identified being male as a significant factor for recovering from CP (OR = 1.48; CI = 1.11–1.99; *p* = 0.008). On the other hand, recovery was less likely for those who were older (OR = 0.83, CI = 0.69–1.00, *p* = 0.050), were overweight (OR = 0.71, CI = 0.52–0.96, *p* = 0.027) or obese (OR = 0.60, CI = 0.40–0.91, *p* = 0.015), had a higher depression score (CI = 0.97, CI = 0.96–1.00, *p* = 0.013) and took pain medications (OR = 0.39, CI = 0.23–0.67, *p* = 0.001) (Figure 2, upper panel).

#### 3.2.2 Developing CP

After the exclusion of subjects with missing values, the model ultimately ran on a sample of 1886 participants (Figure 2). The variables included in the final model as predictors of developing CP (significant results from the univariate analyses) were BMI, depression score, ability to perform daily tasks, feeling tired, sex, taking pain medications, self‐rated general health, sleep quality and smoking (see Table 3B). The multivariable analysis (see Table S6) identified the following significant risk factors for developing chronic pain: being overweight (OR = 1.5, CI = 1.16–1.93, *p* = 0.002) or obese (OR = 1.48, CI = 1.01–2.17, *p* = 0.045) compared to normal weight, a bad sleep quality (OR = 1.06, CI = 1.01–1.11, *p* = 0.015) and being a former smoker (OR = 1.33, CI = 1.02–1.72, *p* = 0.032). On the other hand, male sex (OR = 0.65, CI = 0.51–0.83, *p* = 0.001) was associated with a lower likelihood of developing CP (Figure 2, lower panel).

## 4 DISCUSSION

In a large Swiss cohort (*n* = 4.602), we examined CP trajectory predictors across two follow‐ups over a 5‐year span by analysing biological variables, socio‐demographic characteristics and self‐administered questionnaires on health, depression and sleep. In our cohort, 43.1% (FU1) and 44.4% (FU2) of participants reported CP. Of those with pain at FU2, 72.2% reported mild and only 26.2% moderate or severe intensity (i.e. 11.6% of responders). The only positive predictor for recovery from CP was male sex. Older age, being overweight or obese, higher depression scores and taking pain medications were associated with a lower likelihood of recovering from CP. Risk factors for developing CP were being overweight or obese, having a lower quality of sleep and being a former smoker. Being male was associated with a reduced likelihood of developing CP.

### 4.1 Predictive factors of CP trajectories

Our results align well with known age and sex influences on the prevalence and pathophysiology of pain (Bartley & Fillingim, 2013), with female sex identified in prior studies as a risk factor for developing CP (e.g. Boerner et al., 2018; Eriksen et al., 2004) and younger subjects more likely to recover from CP (Elliott et al., 2002; Eriksen et al., 2004). We confirmed that male sex is a significant factor in CP trajectories, both as a protective factor for development and a predictor of recovery (Eriksen et al., 2004). The implication of sex as a biological factor and gender as a social construct to explain individual differences in pain is an important topic that will hopefully be better considered in future preventive efforts (Keogh & Boerner, 2024; Mogil, 2021).

Prior studies reported that CP prevalence increased proportionally with BMI (Mills et al., 2019). In our study, a normal BMI was associated with both a higher probability of recovery and a lower risk of developing CP, thus confirming prior predictive findings in younger cohorts (Eriksen et al., 2004; Sjøgren et al., 2010). We interpret normal BMI as an indicator of good general health (Ng et al., 2020) and opposed to overweight and obese (which could be associated with illness or sedentarity). Importantly, BMI can be targeted by therapeutic interventions or preventive measures.

Our findings that higher depression scores are linked to lower chances of recovery from CP expand prior results where good mental health predicted recovery (Eriksen et al., 2004). Depression, also assessed with semi‐quantitative interviews, has a documented influence on CP, as shown in cross‐sectional and longitudinal studies (Elliott et al., 2002; Eriksen et al., 2004; Rouch et al., 2023). It is surprising that depression was not a predictor of developing CP, contrary to prior findings, where current and remittent major depressive disorders were associated with the persistence of CP (Von Korff et al., 1993). This could be linked to the multiple co‐variates our model considers since depression was a relevant factor in the univariate analysis. Further congruent considerations from a subsample of the Colaus cohort having undergone in‐depth psychiatric assessments are discussed in a recent publication (Rouch et al., 2023). Yet, in this study, Rouch and colleagues focused specifically on CP and mental health disorders. In contrast, our study includes additional variables such as sleep quality, smoking habits and BMI, which possibly had a stronger influence in the context of the development of CP. Hence, our results suggest that variables related to the psychiatric condition of the patient have a stronger effect on recovery from CP, but the physical general health mitigates these effects in CP development.

Taking pain medications was an adverse predictor for recovery from CP, confirming prior work (Amaya, 2018; Kopec et al., 2004; Sjøgren et al., 2010). Not taking pain medication can be an indicator of low‐grade pain or stronger personal resilience (Esteve et al., 2020). Analgesics might be prescribed more often to people with less cognitive, emotional and care resources, for example, in dementia (Hoffmann et al., 2014) or depression (Jobski et al., 2017). In fact, cognitive impairment was associated with higher likelihood of developing CP in a study on a geriatric subsample of the same cohort (Rouch et al., 2021). Nevertheless, recent research has mechanistically associated the inflammatory process with recovery from acute pain, hence questioning the use of non‐steroidal anti‐inflammatory drugs in this context. Our sample does not encompass subacute pain but could illustrate the risk due to the medication itself (Parisien et al., 2022). Interestingly, adjuvant pain medications were not significantly associated with either recovery or development of CP (univariate analyses). This could be due to their lack of specificity to CP conditions or could further support a specific effect of non‐steroidal anti‐inflammatory medications (the most frequent pain medications prescribed in our cohort).

The directionality and causality of adverse interactions between pain and bad sleep are not resolved and either factor might precipitate a vicious cycle (Andersen et al., 2018; Finan et al., 2013; Selvanathan et al., 2022). Chronic pain impacts sleep quality, with pre‐clinical data highlighting subtle changes in sleep architecture due to increased arousability, even when conventional sleep measures are preserved (Cardis et al., 2021). On the other hand, impairing sleep modifies the threshold of pain sensitivity (Lautenbacher et al., 2006), and a low number of sleep hours has been associated with a higher probability of developing CP (Picavet et al., 2019). Here, we confirm a directional effect of sleep on pain, with a validated measure of sleep quality.

Finally, smokers are more likely to develop CP, compared to non‐ and former smokers (Chris Power et al., 2001; Picavet et al., 2019), also in younger individuals (Alkherayf & Agbi, 2009; Ehrmann Feldman et al., 1999). In our study, we found a higher likelihood of developing CP for former smokers and a non‐significant effect for current smokers. Considering the similar OR parameters and the difference in sample size (i.e. the number of current smokers was about half of the former smokers), we infer that the non‐significant effect for current smokers is most likely due to a smaller statistical power.

### 4.2 Prevalence of chronic pain and neuropathic characteristics

The prevalence of CP (i.e. 43.1% FU1; 44.4% FU2) was similar to recent studies using the same definition (Azevedo et al., 2012; Hagen et al., 2020; Jakobsson, 2010). An earlier study, based on a postal survey found a lower prevalence both in Switzerland (16%) and in Europe (19%) (Breivik et al., 2006), possibly due to their more stringent criteria to define chronic pain. In fact, our definition of CP, that is, pain with a duration of 3 months, regardless of intensity, is in line with the 11th revision of the International Classification of Diseases (ICD‐11) (Treede et al., 2019). On the other hand, moderate (≥5 and <7) and severe (≥7/10) pain affected only a small part of the CP population in our sample (i.e. at FU2: 26.2% of CP+; i.e. 11.6% of responders). This is comparable to the reference article by Breivik et al. (2006) (i.e. 16%), where the definition of CP involved a certain pain intensity (≥5). Age might also be a factor: a study focusing on pain epidemiology in a geriatric (>65 years old) population in Switzerland showed a higher proportion (41%) of intense pain (Cedraschi et al., 2018).

The most common worst painful location was the back, confirming prior findings (Breivik et al., 2006; Elliott et al., 1999; Macfarlane, 2016). Neuropathic characteristics were present in 24% of our CP+ subsample, that is, 11% of the general population, comparably to prior studies (Attal et al., 2011; Torrance et al., 2006; van Hecke et al., 2014). We measured the neuropathic characteristics of CP through a self‐rated questionnaire, which is standard in cohort studies (Attal et al., 2011; Torrance et al., 2006). We observed that CP with neuropathic characteristics is associated with a lower self‐rated general health, lower quality of sleep and more probability of being depressed. Importantly, these effects are present after correcting for pain intensity, confirming prior findings in a contemporary cohort with different, well‐validated measures of depression and sleep. Hence, the sub‐population of individuals with chronic neuropathic pain, who tend to be under‐treated (Torrance et al., 2013) needs to be specifically targeted by therapeutic interventions.

Projected on the population of Switzerland (8.61 million inhabitants (Statista‐Search‐Department, 2022, April)) suggest that >2.5 Mio people in the country have mild CP, about 1 Mio have moderate/severe pain, and >900,000 with neuropathic characteristics. These numbers are important for public health planning and adequate provision of specialized pain centres.

## 5 LIMITATIONS

Only 70.4% of the cohort responded to the pain questionnaire at FU2 (compared to 91.9% at FU1). This reduction could be due to an increase in questionnaire load. When infering the results to the entire Swiss population, one needs to remember that this is an ageing cohort (mean age at FU2 = 62.99 ± 10.46), and the studied subsamples were not entirely representative of the full cohort. The rate of non‐responders was low at FU1 (about 10%) but increased at FU2 (about 30%). A lack of response was more frequent in non‐working individuals, and this was significant at FU2, also congruent with prior literature (Martikainen et al., 2007). This could have led to an under‐estimation of pain prevalence at FU2, as people who are not working could be more likely to have chronic pain. Nevertheless, the predictive models rely on risk/protective factors at FU1, where a reliable representation of non‐working individuals is present, thus we have no reason to believe that this could have brought a bias to our results.

Unfortunately, the basic measure of cognitive function (MMSE) was excluded from the multivariable model due to missing data. Also, pain intensity measures were not available for the first FU, hence, this factor was not included in the predictive model. Nevertheless, the cohort is still followed, with new assessments, holding the promise of including pain intensity and more refined measures of cognitive function in future models.

In sum, this data provides important information for public health prevention endeavours (Jacob et al., 2021). Factors that could be actively targeted to favour a recovery or prevent the development of CP are BMI, sleep quality, smoking and depression. Unfortunately, some vulnerability factors cannot be addressed by interventions (e.g. sex) and challenges come from the interconnected variables (e.g. self‐perceived health). The variables identified here also show some overlap with the ones highlighted through a predictive model of pain evolution that could be further validated in the present cohort (Tanguay‐Sabourin et al., 2023).

## AUTHOR CONTRIBUTIONS

PV, ID, MRS directed data collection; GD, JBR, NF, AD'A, MRS, CB conceived and led the analyses; GD, JBR, MRS, CB wrote the manuscript. All authors discussed the results and commented on the manuscript.

## FUNDING INFORMATION

The CoLaus study was supported by research grants from GlaxoSmithKline, the Faculty of Biology and Medicine of the University of Lausanne, and the Swiss National Science Foundation (grants numbers: 33CSCO‐122661, 33CS30‐139468 and 33CS30‐148401). The funding sources were not involved in the study design, conduct of the study, data collection, analyses of samples or data, interpretation of findings, writing of the report or decision to submit the article for publication. CBR was supported by a Fonds Pépinière from the Faculty of Biology and Medicine of the University of Lausanne and funding from the Fondation Mercier pour la Science. The authors report no conflict of interest.

## CONFLICT OF INTEREST STATEMENT

None.

## Supporting information


Data S1.
