# Supplementary material for: Correlates of chronic pain onset and recovery in the CoLaus cohort
Source: Eur J Pain. 2024 Aug 7;29(2):e4712. doi: 10.1002/ejp.4712 (PMC11671331; doi:10.1002/ejp.4712)
Supplement: Supplementary file 1 — Data S1. [file EJP-29-0-s001.docx]

### Supplementary materials


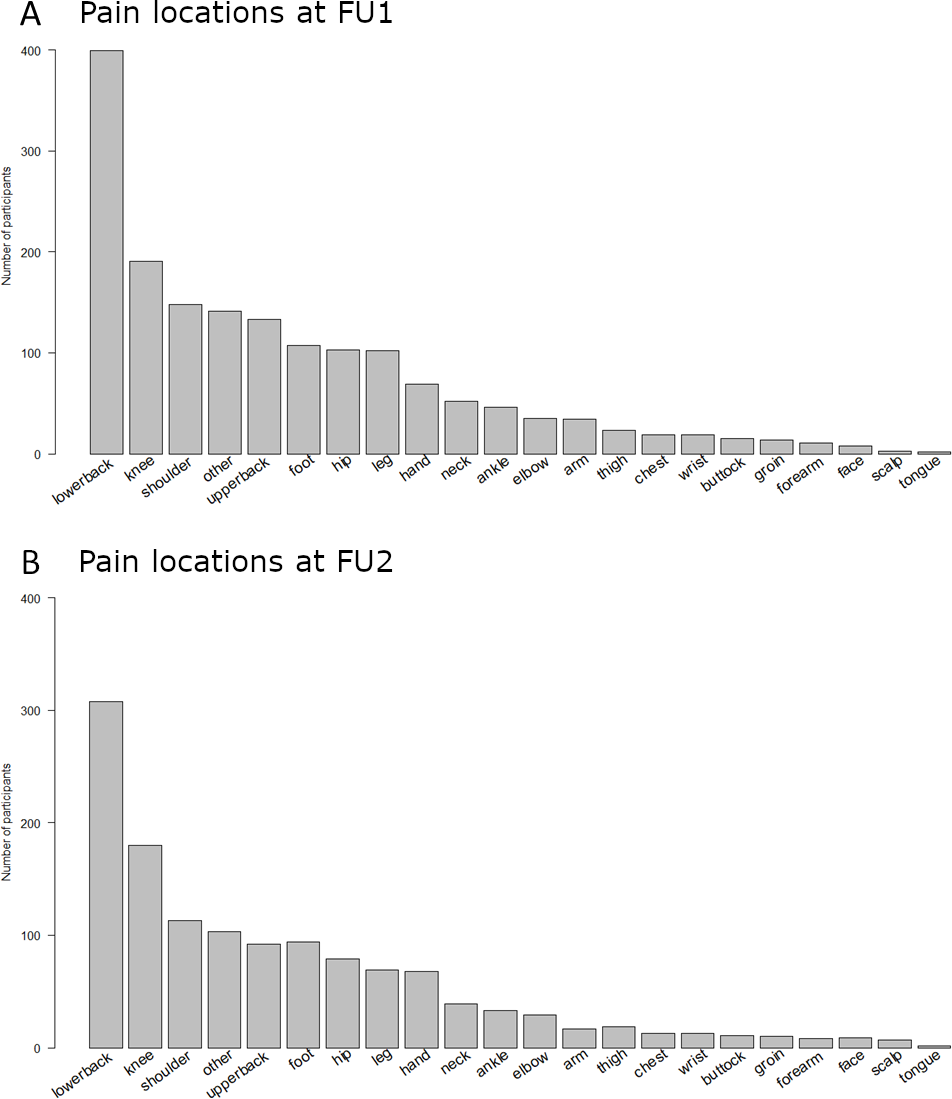


***Figure S1: Pain location. Pain location at FU1 and FU2****. At each follow-up, CP+ participants selected the most painful location from a list.*


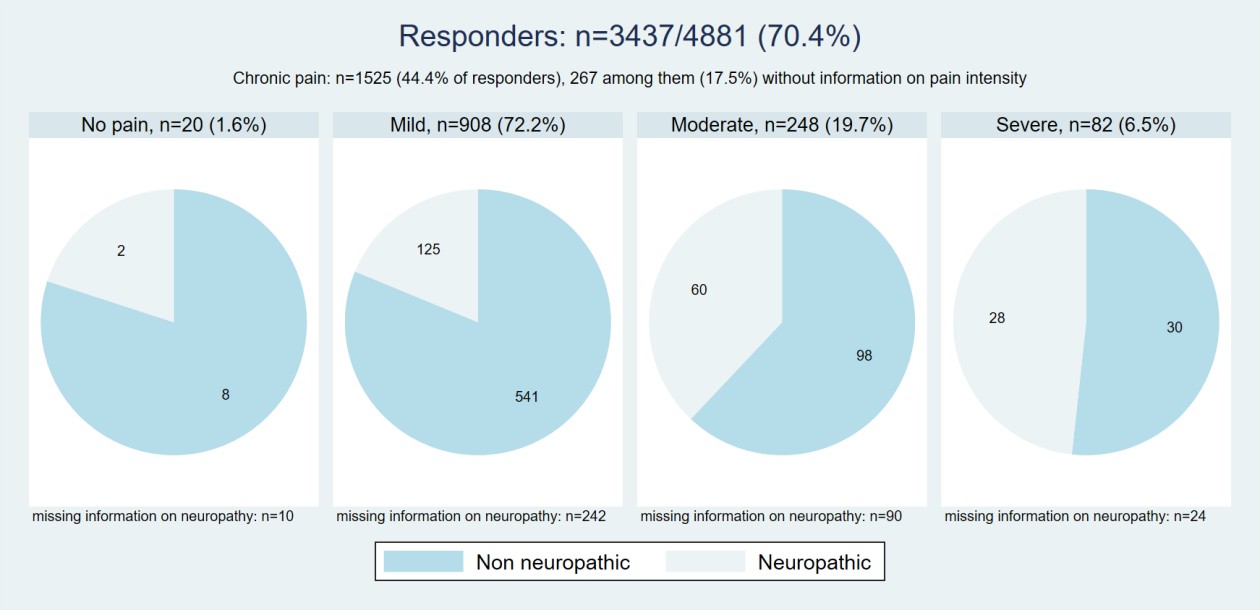


***Figure S2: Schematic representation of the cohort.*** *Description of the CP subsample characteristics at FU2: From FU2, CP+ participants answered to questions on pain intensity (categorized as mild, moderate and severe) and type (neuropathic vs not neuropathic). Number and percentage of participants responding to the pain questionnaire, reporting CP and proportion of individuals with neuropathic pain per intensity category are represented.*

| Pain Medication | Opioids | Methadone |
| --- | --- | --- |
|  |  | Buprenorphine |
|  |  | Dihydrocodeine |
|  |  | Tapentadol |
|  |  | Morphine |
|  |  | Fentanyl |
|  |  | Oxycodone combined |
|  |  | Oxycodone |
|  |  | Codeine combined |
|  |  | Tramadol |
|  |  | Tramadol combined |
|  | NSAID / Paracetamol | Meloxicam |
|  |  | Naproxen |
|  |  | Tenoxicam |
|  |  | Piroxicam |
|  |  | Indometacin |
|  |  | Dexibuprofen |
|  |  | Dexketoprofen |
|  |  | Lornoxicam |
|  |  | Nimesulide |
|  |  | Etodolac |
|  |  | Diclofenac combined |
|  |  | Ketoprofen |
|  |  | Etoricoxib |
|  |  | Acemetacin |
|  |  | Mefenamic acid |
|  |  | Celecoxib |
|  |  | Diclofenac |
|  |  | Ibuprofen |
|  |  | Paracetamol |
|  |  | Sodium metamizole |
| Pain Medication Adjuvants | Gabapentinoids | Gabapentin |
|  |  | Pregabalin |
|  | Tricyclic antidepressants & NSRI | Dibenzepin |
|  |  | Doxepin |
|  |  | Reboxetine |
|  |  | Opipramol |
|  |  | Nortriptyline |
|  |  | Trimipramine |
|  |  | Clomipramine |
|  |  | Amitriptyline |
|  |  | Venlafaxine |
|  |  | Duloxetine |

***Table S1: Considered list of Pain medication, with the categories they were attributed to.***

*NSAID: non-steroidal anti-inflammatory drugs; NSRI: noradrenaline serotonin reuptake inhibitors.*

| **Variable** | **No CP**  **(n = 20)** | **Mild CP**  **(n = 908)** | **Moderate or severe CP**  **(n = 330)** | ***p-value*** |
| --- | --- | --- | --- | --- |
| **Sex** |  |  |  | <0.001** |
| **woman** | 7 (35.0%) | 528 (58.1%) | 227 (68.8%) |  |
| **man** | 13 (65.0%) | 380 (41.9%) | 103 (31.2%) |  |
| **Age (years) - mean (sd)** | 60.4 (11.4) | 61.9 (9.9) | 64.3 (10.5) | <0.001** |
| **Working** |  |  |  | <0.001** |
| **yes** | 13 (65.0%) | 525 (58.3%) | 140 (42.8%) |  |
| **no** | 7 (35.0%) | 375 (41.7%) | 187 (57.2%) |  |
| ***missing*** | 0 | 8 | 3 |  |
| **Living situation** |  |  |  | 0.265 |
| **living alone** | 4 (20.0%) | 252 (28.3%) | 103 (31.8%) |  |
| **single parent family** | 1 (5.0%) | 63 (7.1%) | 25 (7.7%) |  |
| **couple without children** | 8 (40.0%) | 326 (36.5%) | 128 (39.5%) |  |
| **couple with children** | 7 (35.0%) | 251 (28.1%) | 68 (21.0%) |  |
| ***missing*** | 0 | 16 | 6 |  |
| **How would you rate your health** |  |  |  |  |
| **mean (sd)** | 1.7 (0.5) | 2.1 (0.7) | 2.5 (0.8) | <0.001** |
| ***missing*** | 0 | 1 | 0 |  |
| **Sleep - being a bad sleeper** |  |  |  | <0.001** |
| **no** | 11 (84.6%) | 410 (62.4%) | 81 (41.3%) |  |
| **yes** | 2 (15.4%) | 247 (37.6%) | 115 (58.7%) |  |
| ***missing*** | 7 | 251 | 134 |  |
| **Depression (ces-d)** |  |  |  | <0.001** |
| **not depressed** | 20 (100.0%) | 749 (85.2%) | 232 (75.1%) |  |
| **depressed** | 0 (0.0%) | 130 (14.8%) | 77 (24.9%) |  |
| ***missing*** | 0 | 29 | 21 |  |

***Table S2: Characteristics of the people with Chronic pain (CP+), when classified into pain intensity subsamples.***

*The last column per each follow-up indicates the p-value of the Chi-squared or ANOVA expressing the difference between groups. ** indicates p-values <.001 and * indicates p-values <.01.* Additional OR test was run when a significant difference was found, which highlighted only one large difference: a higher proportion of non-working individuals in the Moderate/severe group (OR= 2.02; CI=1.52; 2.69).

**Variable FU2 Neuropathic CP (N = 226)**

**FU2 Non neuropathic CP (N = 699)**

**p-value**

| **Sex** |  |  | 0.153 |
| --- | --- | --- | --- |
| **woman** | 145 (64.2%) | 411 (58.8%) |  |
| **man** | 81 (35.8%) | 288 (41.2%) |  |
| **Age (years) - mean (sd)** | 61.5 (10.0) | 62.4 (9.8) | 0.275 |
| **Working** |  |  | 0.145 |
| **yes** | 114 (50.7%) | 388 (56.2%) |  |
| **no** | 111 (49.3%) | 302 (43.8%) |  |
| ***missing*** | 1 | 9 |  |
| **Living situation** |  |  | 0.023 |
| **living alone** | 75 (33.6%) | 193 (28.1%) |  |
| **single parent family** | 23 (10.3%) | 40 (5.8%) |  |
| **couple without children** | 75 (33.6%) | 259 (37.8%) |  |
| **couple with children** | 50 (22.4%) | 194 (28.3%) |  |
| ***missing*** | 3 | 13 |  |
| **How would you rate your health** |  |  |  |
| **mean (sd)** | 2.6 (0.8) | 2.1 (0.7) | <0.001** |
| ***missing*** | 0 | 1 |  |
| **Sleep - being a bad sleeper** |  |  | <0.001** |
| **no** | 62 (42.2%) | 309 (61.4%) |  |
| **yes** | 85 (57.8%) | 194 (38.6%) |  |
| ***missing*** | 79 | 196 |  |
| **Depression (ces-d)** |  |  | <0.001** |
| **not depressed** | 145 (66.5%) | 580 (85.9%) |  |
| **depressed** | 73 (33.5%) | 95 (14.1%) |  |
| ***missing*** | 8 | 24 |  |

***Table S3: Neuropathic and non-neuropathic pain.*** *Characteristics of the CP+ sample, grouped by pain type: at FU2, participants who reported CP were grouped in subsample of either neuropathic or non neuropathic pain (derived from the score of the DN4 questionnaire). The subsample characteristics are shown (variables listed in the first column) in terms of number of participants and percentage over the CP+ subsample.*

*The last column per each follow-up indicates the Chi-squared or t-test parameter expressing the difference between groups. ** indicates p-values <.001 and * indicates p-values <.01.*

***Table S4****:* ***Pain medications****.*

**FU1 CP+ *(1*’*985)* FU2 CP+ *(1*’*525)***

|  | n *(%)* | n *(%)* |
| --- | --- | --- |
| **Opioids** | 52 *(2,6)* | 71 *(4.7)* |
| **NSAID & paracetamol** | 229 *(11.5)* | 332 *(21.8)* |
| **Antidepressants: tricyclic & NSRI** | 66 *(3.3)* | 38 *(2.5)* |
| **Gabapentinoids** | 20 *(1.0)* | 27 *(1.8)* |

*NSAID:* non-steroidal anti-inflammatory drugs; NSRI: noradrenaline serotonin reuptake inhibitors.

***Table S5: Results of the recovering from the*** *multivariable* ***model for recovery from chronic pain***

| **Variable** | **Odds Ratio (95% CI; p-value)** |
| --- | --- |
| **Sex** |  |
| Woman | 1 (ref.) |
| Man | 1.484 (1.107 - 1.989; p=0.008) |
| **Age (in decades)** | 0.829 (0.688 - 1.000; p=0.050) |

| **How would you rate your health** | 0.789 (0.622 - 1.001; p=0.051) |
| --- | --- |
| **Felt tired** | 0.918 (0.718 - 1.174; p=0.494) |
| **Difficulties in taking a shower or dressing up** | 1.053 (0.661 - 1.677; p=0.827) |
| **Difficulties in going shopping our doing household tasks** | 0.616 (0.374 - 1.015; p=0.057) |
| **CES-D overall score** | 0.974 (0.955 - 0.995; p=0.013) |
| **PSQI** | 1.000 (0.953 - 1.050; p=0.991) |
| **BMI Category** |  |
| Normal | 1 (ref.) |
| Overweight | 0.706 (0.519 - 0.960; p=0.027) |
| Obese | 0.604 (0.401 - 0.908; p=0.015) |
| **Alcohol (Yes vs No)** | 1.092 (0.745 - 1.602; p=0.652) |
| **Social financial help (Yes vs No)** | 0.748 (0.519 - 1.078; p=0.119) |
| **Working (Yes vs No)** | 0.818 (0.570 - 1.176; p=0.279) |
| **Pain medications (Yes vs No)** | 0.388 (0.226 - 0.665; p=0.001) |

*.*

| **Variable** | **Odds Ratio (95% CI; p-value)** |
| --- | --- |
| **Sex** |  |
| Woman | 1 (ref.) |
| Man | 0.650 (0.510 - 0.829; p=0.001) |
| **How would you rate your health** | 1.210 (0.981 - 1.493; p=0.075) |
| **Felt tired** | 1.132 (0.916 - 1.399; p=0.252) |
| **Difficulties in going shopping or doing household tasks** | 1.176 (0.713 - 1.939; p=0.525) |
| **CES-D overall score** | 1.004 (0.987 - 1.021; p=0.631) |
| **PSQI** | 1.059 (1.011 - 1.110; p=0.015) |
| **BMI Category** |  |
| Normal | 1 (ref.) |
| Overweight | 1.495 (1.159 - 1.928; p=0.002) |
| Obese | 1.477 (1.009 - 2.161; p=0.045) |
| **Smoking status** |  |
| Non-smoker | 1 (ref.) |
| Former smoker | 1.326 (1.024 - 1.718; p=0.032) |
| Current smoker | 1.241 (0.904 - 1.705; p=0.182) |
| **Pain medications (Yes vs No)** | 2.169 (0.932 - 5.050; p=0.072) |

***Table S6: Results of the developing CP model.*** *Results from multivariable model of risk factors for developing CP: to assess the variables associated with developing CP, we first computed univariate analyses with each variable of the database regressed against developing CP (yes/ no). We then computed a multivariable model including only the variables with significant results from the univariate analysis. This table shows the variables included in the multivariable model and the results for each of them in terms of p-value, estimated parameter and odd ratio. See Table 3 B for univariate analyses’ results.*

Table S7: Summary table of missing values

|  |  | FU 2 pain questionnaire | | |  |
| --- | --- | --- | --- | --- | --- |
|  |  | **Non-responder** | **Responder** | **missing** | **Total** |
| FU1 pain quest. | **Non-responder** | 199 | 153 | 110 | **462** |
|  | **Responder** | 922 | *3060* | 620 | **4602** |
|  | **missing** | 323 | 224 | 0 | **547** |
|  | **Total** | **1444** | **3437** | **730** | **5611** |

Table S8: Missing data, detailed report for each variable with 1 or more missing data

| Variable at FU1 | Number of deleted observations | Number of observations remaining in the dataset |
| --- | --- | --- |
| Patient in FU1 dataset | 0 | 5064 |
| Patient in FU2 dataset | 730 | 4334 |
| How would you rate your health | 25 | 4309 |
| CES-D overall score | 596 | 3713 |
| BMI categories | 27 | 3686 |
| Do you currently drink alcohol | 7 | 3679 |
| Smoking status | 5 | 3674 |
| Do you receive social help | 12 | 3662 |
| Occupational position | 16 | 3646 |
| What is your current situation | 4 | 3642 |
| PSQI score | 384 | 3258 |
| Diabetes | 11 | 3247 |
